# An Entropy Regularization *k*-Means Algorithm with a New Measure of between-Cluster Distance in Subspace Clustering

**DOI:** 10.3390/e21070683

**Published:** 2019-07-12

**Authors:** Liyan Xiong, Cheng Wang, Xiaohui Huang, Hui Zeng

**Affiliations:** School of Information Engineering Department, East China Jiaotong University, R.d 808, East Shuanggang Avenue, Nanchang 330013, China

**Keywords:** k-means, between-cluster information, entropy regularization, data mining

## Abstract

Although within-cluster information is commonly used in most clustering approaches, other important information such as between-cluster information is rarely considered in some cases. Hence, in this study, we propose a new novel measure of between-cluster distance in subspace, which is to maximize the distance between the center of a cluster and the points that do not belong to this cluster. Based on this idea, we firstly design an optimization objective function integrating the between-cluster distance and entropy regularization in this paper. Then, updating rules are given by theoretical analysis. In the following, the properties of our proposed algorithm are investigated, and the performance is evaluated experimentally using two synthetic and seven real-life datasets. Finally, the experimental studies demonstrate that the results of the proposed algorithm (ERKM) outperform most existing state-of-the-art *k*-means-type clustering algorithms in most cases.

## 1. Introduction

Clustering is a process of dividing a set of points into multiple clusters. In this process, the similarity among points in a cluster is higher than that among points from different clusters. Due to high efficiency, *k*-means-type clustering algorithms have been widely used in various fields of real life [1,2], such as marketing [3] and bioinformatics [4].

In the past few decades, the research of clustering techniques has been extended to many fields [5] such as gene analysis [6] and community detection [7]. The idea of most clustering algorithms aims at making the similarity among points in a cluster higher than those from different clusters, namely to minimize the within-cluster distance [8]. However, the traditional clustering methods seem to be weak when dealing with high-dimensional data under many cases [9]. For example, for points in the same cluster, the distance between each dimension should be small, but the true results after clustering by traditional clustering algorithms are that the distance between some dimensions may be very large.

The classic *k*-means-type algorithms cannot automatically determine the importance of a dimension, i.e., which are important dimensions and which are noise dimensions, because they treat all dimensions equally in the clustering process [10]. At the same time, it is also worth noting that the valid dimensions are often a part of the dimensions rather than all in the process of high-dimensional data clustering. Fortunately, in recent years, some subspace clustering algorithms [11,12,13,14,15,16] have largely alleviated this problem. For example, the work in [16] considered that different dimensions make different contributions, by introducing entropy weighting to the identification of objects in clusters.

To date, many subspace clustering algorithms have been proposed and well applied in various fields; however, most of them overlooked the within-cluster distance in the clustering process. Namely, the between-cluster information is not fully utilized [17] for improving the clustering performance. The between-cluster information is utilized in some algorithms [5,18,19]. However, in some cases, e.g., clusters with a blurred decision boundary, the existing methods cannot conquer this effectively.

**Figure 1:**
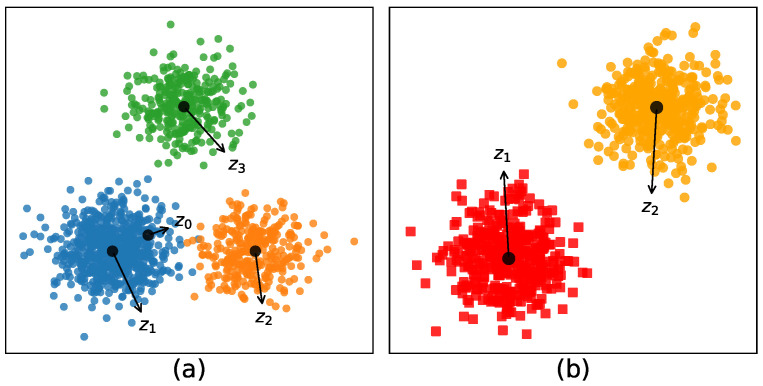
(**a**) The idea with the global center; (**b**) the idea without the global center.

In traditional ways, many algorithms utilize the between-cluster information by introducing the global center [5,18]. The main idea of these methods is to maximize the distance between the center of each cluster and the global center. Under these circumstances, if the number of points in each cluster differs greatly, then the global center will be heavily biased toward the cluster with a large number of points (e.g., in Figure 1a, the global center $z_{0}$ is heavily biased toward Cluster 1). Subsequently, in order to maximize the distance between $z_{0}$ and $z_{1}$, the latent center of Cluster 1 will greatly deviate from its true cluster center $z_{1}$, resulting in the decrease of the performance in convergence.

Motivated by this, we propose a new measure of between-cluster distance based on entropy regularization in this paper, which is totally different from these existing methods (see, for instance, [5,18,19,20,21,22]). The key idea is to maximize the distance between the points in the subspace that do not belong to the cluster and the center point of the cluster. As shown in Figure 1b, ERKM maximizes the distance between $z_{1}\left(z_{2}\right)$ and circle-points (square-points) in subspace. In order to award more dimensions to make contributions to the identification of each cluster, avoiding few sparse dimensions, we also introduce the entropy regularization term in the objective function.

Moreover, the measure of between-cluster distance proposed in this paper can also be applied to other clustering algorithms, not only limited to *k*-means-type algorithms. The main contributions of this paper are as follows:

- The study on subspace clustering mentioned in previous papers is summarized.
- A new *k*-means clustering algorithm combining between-cluster distance and entropy regularization [16,23] is introduced.
- By optimization, the update rules of ERKM algorithm are obtained, and the convergence and robustness analysis of the algorithm are given.
- The hyperparameters are studied on synthetic and real-life datasets. Finally, through experimental comparison, the results show that the ERKM algorithm outperforms most existing state-of-the-art *k*-means-type clustering algorithms.

The rest of this paper is organized as follows: Section 2 is an overview of related works about subspace clustering algorithms. Section 3 presents a new *k*-means-type subspace clustering algorithm, and update rules are given. Experiments and results are analyzed in Section 4. In Section 5, conclusions and future work are presented.

## 2. Related Work

The research on subspace clustering [24] has always been an important direction in clustering algorithms. In order to achieve high performance, many methods, such as sparse clustering-based methods [25,26,27], weight entropy-based methods [16,28], between-cluster information-based methods [29,30,31], and so on, have been proposed. Furthermore, different ways in different cases will have a different impact on the results of clustering algorithms. In this section, we will give a brief summary of the ways in different fields proposed in recent years.

For a clear statement of related work, we first introduce the most common notations. Let $X=\left(x_{ij}\right)\in R^{n\times m}$ denote a dataset in the matrix form with *n* points and *m* dimensions. $U=\left(u_{li}\right)\in R^{k\times n}$ denotes a partition matrix, where $u_{li}=1$ indicates that point *i* is assigned to cluster *l*; otherwise, it is not assigned to cluster *l*. $Z=\left(z_{lj}\right)\in R^{k\times m}$ is a set of *k* vectors representing the centers of *k* clusters. *W* is a weighting matrix or vector depending on specific algorithms.

### 2.1. Sparseness-Based Methods

In order to cluster high-dimensional objects in subspace, Witten et al. [26] proposed a novel framework for sparse clustering, which clusters the points using an adaptively-chosen subset of the dimensions. The main idea it to use a lasso-type penalty to select the features. This framework can be used for sparse *k*-means clustering and also sparse hierarchical clustering. Taking sparse *k*-means as an example, its objective function can be reformulated as follows:

(1) $$P\left(U,Z,W\right)=\underset{u_{1},\ldots ,u_{k},W}{\mathrm{maximize}}\left\{\sum_{j=1}^{m}w_{j}\left(\frac{1}{n}\sum_{i=1}^{n}\sum_{i^{\prime }=1}^{n}{\left(x_{ij}-x_{i^{\prime }j}\right)}^{2}-\sum_{p=1}^{k}\frac{1}{n_{p}}\sum_{i,i^{\prime }\in z_{p}}{\left(x_{ij}-x_{i^{\prime }j}\right)}^{2}\right)\right\},$$

subject to:

$${∥W∥}^{2}\leq 1,\;\;\;{∥W∥}_{1}\leq s,\;\;\;w_{j}\geq 0\;\forall j.$$

The weights in this algorithm will be sparse for an appropriate choice of the tuning parameter *s*, which should satisfy $1\leq s\leq \sqrt{m}$. If $w_{1}=,\cdots ,=w_{p}$, the objective function (1) simply reduces to the criterion standard *k*-means clustering.

Combining the penalized likelihood approach with an $L_{1}$ penalty function and model-based clustering [32], Pan et al. [27] presented a penalized model-based clustering algorithm, automatically selecting features and delivering a sparse solution. This can be used for high-dimensional data. Its objective function can be expressed as follows:

(2) $$\log L_{c,P}\left(W\right)=\sum_{p=1}^{k}\sum_{i=1}^{n}z_{pi}\left[\log\pi_{p}+\log f_{p}\left(x_{i};\theta_{p}\right)\right]-h_{\lambda }\left(W\right),$$

where $h_{\lambda }\left(\cdot \right)$ is a penalty function with penalization parameter λ. The choice of $h_{\lambda }\left(\cdot \right)$ depends on the goal of the analysis.

### 2.2. Entropy-Based Methods

For the sake of stimulating dimensions in subspace, Jing et al. [16] extended the *k*-means algorithm to calculate a weight for each dimension in each cluster, achieved by including the weight entropy in the objective function, and used it to identify the subsets of important dimensions. The weights of all dimensions can be automatically computed only by adding an additional step to the *k*-means clustering process. The objective function of this algorithm (EWKM) can be written as follows:

(3) $$P\left(U,Z,W\right)=\sum_{p=1}^{k}\left[\sum_{i=1}^{n}\sum_{j=1}^{m}u_{pi}w_{pj}{\left(z_{pj}-x_{ij}\right)}^{2}+\gamma \sum_{j=1}^{m}w_{pj}logw_{pj}\right],$$

subject to:

$$\left\{\begin{array}{c} \sum_{p=1}^{k}u_{pi}=1,\;\;1\leq i\leq n,\;\;1\leq p\leq k,\;\;u_{pi}\in \left\{0,1\right\},\\ \sum_{j=1}^{m}w_{pj}=1,\;\;1\leq p\leq k,\;\;1\leq j\leq m,\;\;0\leq w_{pj}\leq 1, \end{array}\right.$$

where the weight $w_{lj}$ is to measure the contribution (or importance) of the *j*-th dimension in cluster *l*. The more $w_{lj}$, the higher contribution of dimension *j* in partitioning cluster *l*. The positive parameter γ controls the strength of the incentive for clustering on more dimensions. The first term in (3) is the sum of the within-cluster distance and the second term the negative weight entropy, which is to stimulate more dimensions to contribute to the identification of clusters, avoiding the problem of identifying clusters by a few dimensions.

To achieve the same goal, Zhou et al. [28] proposed a novel fuzzy *c*-means algorithm, by using a weighted dissimilarity measure and adding a weight entropy regularization term to the objective function. It only adds a fuzzy index to the algorithm EWKM, and its objective function can be re-written as follows:

(4) $$P\left(U,Z,W\right)=\sum_{p=1}^{k}\left[\sum_{i=1}^{n}\sum_{j=1}^{m}u_{pi}^{\alpha }w_{pj}{\left(z_{pj}-x_{ij}\right)}^{2}+\gamma \sum_{j=1}^{m}w_{pj}logw_{pj}\right],$$

where the exponent α≥1 is to control the extent of membership sharing between the fuzzy clusters. When α=1, then the objective function (4) simply reduces to the EWKM algorithm.

### 2.3. Between-Cluster Measure-Based Methods

#### 2.3.1. Liang Bai’s Methods

The *k*-modes algorithms and its modified versions for categorical data have always been a hot topic in clustering algorithms. Like the clustering algorithm for real number data, the use of between-cluster information is also particularly vital for the categorical data. A good between-cluster measure approach can often achieve better results.

Based on the fuzzy *k*-modes algorithm [1], Bai et al. [21] presented a new algorithm by adding the between-cluster information term so that one can simultaneously minimize the within-cluster dispersion and enhance the between-cluster separation. The objective function can be written as follows:

(5) $$\begin{array}{c} F_{n}\left(U,Z,\gamma \right)=\sum_{p=1}^{k}\sum_{i=1}^{n}u_{pi}^{\alpha }\sum_{j=1}^{m}\delta \left(z_{pj},x_{ij}\right)+\gamma \sum_{p=1}^{k}\sum_{i=1}^{n}u_{pi}^{\alpha }\frac{1}{n}\sum_{i=1}^{n}s\left(z_{p},x_{i}\right), \end{array}$$

where the second term in (5) is a definition of the between-cluster information, which is a similarity measure between cluster $z_{l}$ and point $x_{i}$ and defined as:

$$s\left(z_{p},x_{q}\right)=\sum_{j=1}^{m}\phi \left(z_{pj},x_{qj}\right),\;\;\phi \left(z_{pj},x_{qj}\right)=\left\{\begin{array}{c} 1,\;\;\;z_{pj}=x_{qj}\\ 0,\;\;\;z_{pj}\neq x_{qj} \end{array},\right.$$

where the parameter γ is used to maintain a balance between the effect of the within-cluster information and the between-cluster information on the minimization process of the objective function (5).

Similar to (5), Bai et al. [22] defined another between-cluster similarity term to evaluate the between-cluster separation. The between-cluster similarity term is defined as:

(6) $$B_{g}\left(U,Z\right)=\sum_{p=1}^{k}\sum_{i=1}^{n}u_{pi}\frac{1}{n}\sum_{p=1}^{n}s_{g}\left(z_{p},x_{q}\right),$$

where $s_{g}\left(z_{p},x_{i}\right)$ is a similarity measure between $z_{p}$ and $x_{i}$. At the same time, this term with different forms can be added in different *k*-means-types algorithm in different cases, such as in (7).

(7) $$F_{0}\left(W,Z,\gamma \right)=\sum_{p=1}^{k}\sum_{i=1}^{n}u_{pi}d_{0}\left(z_{p},x_{i}\right)+\gamma B_{0}\left(U,Z\right),$$

where γ≥0 is to maintain a balance between the effect of the within-cluster information and the between-cluster information on the minimization process. $B_{0}$ is defined as:

$$B_{0}\left(U,Z\right)=\sum_{p=1}^{k}\sum_{i=1}^{n}u_{pi}\frac{1}{n}\sum_{p=1}^{n}\left(m-d_{0}\left(z_{p},x_{q}\right)\right),$$

where $d_{0}\left(z_{p},x_{q}\right)$ is the distance between cluster $z_{p}$ and point $x_{q}$.

#### 2.3.2. Huang’s Methods

*k*-means-type clustering aims at partitioning a dataset into clusters such that the objects in a cluster are compact and in different clusters are well separated. By integrating within-cluster compactness and between-cluster separation, Huang et al. [18] also designed a new method to utilize the between-cluster information. Based on this basic idea, many traditional algorithms without considering between-cluster separation can be modified, such as basic *k*-means and wk-means [10] algorithms.

An example of a modified objective function of *k*-means algorithm can be re-written as follows:

(8) $$P\left(U,Z\right)=\sum_{p=1}^{k}\sum_{i=1}^{n}u_{pi}\sum_{j=1}^{m}\frac{{\left(x_{ij}-z_{pj}\right)}^{2}}{{\left(z_{pj}-z_{0j}\right)}^{2}},$$

where $z_{0j}$ is the *j*-th feature of the global center $z_{0}$ of a dataset. Its main idea is to minimize the distances between objects and the center of the cluster that the objects belong to, while maximizing the distances between centers of clusters and the global center. From (8), we can find that it considers the distance between cluster center $z_{p}$ and global center $z_{0}$, indicating that the greater the distance, the higher the probability of cluster center $z_{p}$ being confirmed.

Based on a similar motivation, Huang et al. [19] proposed another new discriminative subspace *k*-means-type clustering algorithm, which integrates the within-cluster compactness and the between-cluster separation simultaneously. Its main idea is to use a three-order tensor weighting method to discriminate the weights of features when comparing every pair of clusters. The objective function can be represented as:

(9) P(U,W,Z)=∑p=1k∑q=1q≠pk∑j=1mwpqjDpqj+γ∑p=1k∑q=1q≠pk∑j=1mwpqjlog(wpqj),Dp,q,j=∑i=1nuip[(xij−zpj)2−η(zpj−zqj)2],

where the weight *w* is a three-order tensor and each value $w_{p,q,j}$ in denotes the importance of the feature *j* in cluster *p* when comparing cluster *p* to cluster *q*, where p≠q. γ is a parameter that controls the distribution of the weight, and parameter η is used for balancing the effect of within-cluster compactness and between-cluster separation. In the objective function (9), the first term includes two parts: one is the sum of within-cluster compactness; the other is the sum of the distances between centers of different clusters, which involves maximizing the inter-cluster separation.

### 2.4. Others

While between-cluster information and weight entropy are commonly used in most subspace clustering algorithms, their performance can be further enhanced. A major weakness of entropy-based clustering algorithms is that they do not consider the between-cluster information. Motivated by this, Deng et al. [5] proposed an Enhanced Soft Subspace Clustering (ESSC) algorithm, combining the EWKM and a new measure between-cluster distance, which is to maximize the distance between cluster centers and global center in subspace.

**Figure 2:**
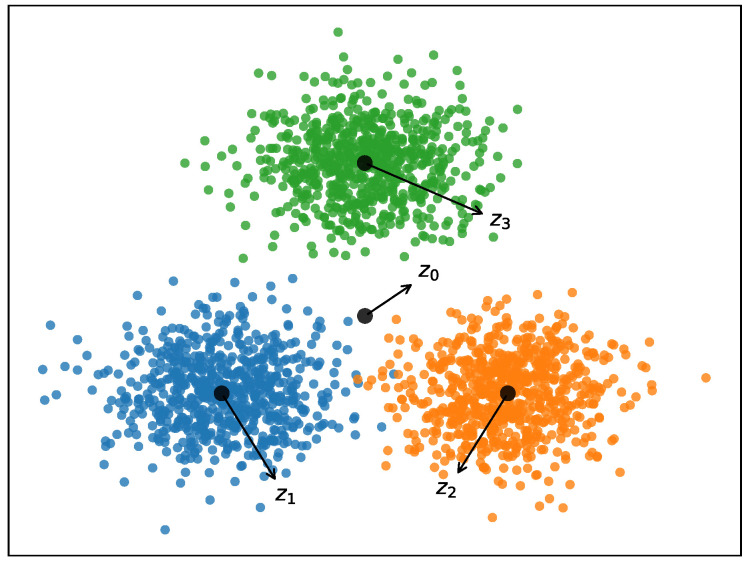
Main idea of ESSC ($z_{1},z_{2},z_{3}$ are the cluster centers, and $z_{0}$ is the global center). ESSC: Enhanced Soft Subspace Clustering.

An intuitive explanation of the between-cluster separation is given below. As illustrated in Figure 2, the main idea of ESSC is to both minimize the with-cluster distance and maximize between-cluster distance (e.g., $\left|\right|z_{0}z_{1}\left||,|\right|z_{0}z_{2}\left||,|\right|z_{0}z_{3}\left|\right|$) simultaneously in subspace, which can make the three clusters’ centers $v_{1},v_{2},v_{3}$ as far apart as possible from each other.

The objective function of ESSC can be expressed as:

(10) $$\begin{array}{c} P\left(U,Z,W\right)=\sum_{p=1}^{k}\sum_{i=1}^{n}u_{pi}^{\beta }\sum_{j=1}^{m}w_{pj}{\left(x_{ij}-z_{pj}\right)}^{2}+\\ \gamma \sum_{p=1}^{k}\sum_{j=1}^{m}w_{pj}\log w_{pj}-\alpha \sum_{p=1}^{k}\left(\sum_{i=1}^{n}u_{pi}^{\beta }\right)\sum_{j=1}^{m}w_{pj}{\left(z_{pj}-z_{0j}\right)}^{2} \end{array}$$

where β and $W=[W_{1},\cdots ,W_{k}]$ are the fuzzy index and weighting matrix, respectively, and $W_{q}=\left[w_{q1},\cdots ,w_{qm}\right]$, $w_{qj}$ is the weight of the *j*-th feature in the *p*-th cluster. γ>0,α>0 are two parameters. $z_{0}$ is the global center of all points. The total weighted distance in the subspace between the global center and each cluster center is calculated by the third term in (10), which is used to maximize the between-cluster distance as much as possible in clustering.

Enlightened by the regularization, Chang et al. [33] proposed a novel Fuzzy *c*-Means (FCM) model with sparse regularization, by reformulating the FCM objective function into the weighted between-cluster sum of squares form and imposing the spare regularization on the weights. Its objective function can be rewritten as follows:

(11) $$P\left(U,Z,W\right)=\mathrm{maximize}\left\{\sum_{j=1}^{m}w_{j}\left(\frac{1}{n}\sum_{i=1}^{n}\sum_{i^{\prime }=1}^{n}{\left(x_{ij}-x_{i^{\prime }j}\right)}^{2}-\sum_{p=1}^{k}\frac{1}{n_{p}}\sum_{i=1}^{n}\sum_{i^{\prime }=1}^{n}u_{ip}^{\alpha }u_{i^{\prime }p}^{\alpha }{\left(x_{ij}-x_{i^{\prime }j}\right)}^{2}\right)\right\},$$

subject to:

$$\left\{\begin{array}{c} \sum_{p=1}^{k}u_{ip}=1,0\leq u_{ip}\leq 1,\\ {∥W∥}_{2}\leq 1,{∥W∥}_{q}^{q}\leq s,\\ w_{j}\geq 0,j=1,\ldots ,m, \end{array}\right.$$

where 0<q≤1, ${∥W∥}_{q}^{q}=\sum_{j=1}^{m}{\left|w_{j}\right|}^{q}$, and ${∥W∥}_{q}^{q}$ is the sparse regularization constraint conditions, which is to make the weight of some dimensions near zero so that the relevant features can be found.

## 3. Entropy Regularization Clustering

The ERKM algorithm proposed in this paper mainly extends the EWKM algorithm [16]. On this basis, a new method of measuring between-cluster distance is introduced. The idea of the new method is to maximize the between-cluster distance by maximizing the distance between the center of a cluster and the points that do not belong to the cluster in subspace. The new algorithm uses vector-weighting to find the best subspace and adjusts the weight of each dimension through the entropy regularization term. Based on this idea, we firstly develop an objective function for the algorithm. Then, the update rules of each variable are obtained by minimizing the objective function, and the convergence is proven.

### 3.1. ERKM Algorithm

Let $W=\{w_{1},w_{2},\cdots ,w_{m}\}$ be the weights of features that represent the contribution of each dimension in the clustering process.

The new objective function is written as follows:

(12) $$\begin{array}{c} P\left(W,U,Z\right)=\sum_{p=1}^{k}\sum_{i=1}^{n}u_{pi}\sum_{j=1}^{m}w_{j}{\left(x_{ij}-z_{pj}\right)}^{2}\\ +\gamma \sum_{j=1}^{m}w_{j}\log w_{j}-\eta \sum_{p=1}^{k}\sum_{i=1}^{n}\left(1-u_{pi}\right)\sum_{j=1}^{m}w_{j}{\left(x_{ij}-z_{pj}\right)}^{2}, \end{array}$$

subject to:

$$\left\{\begin{array}{c} \sum_{j=1}^{m}w_{j}=1,\;\;0<w_{j}<1,\\ \sum_{p=1}^{k}u_{pi}=1,\;\;u_{pi}\in \left\{0,1\right\}. \end{array}\right.$$

There are three terms in the objective function: the weighted within-cluster distance term, the entropy regularization term, and the weighted between-cluster distance term. The first and second terms are directly extended from EWKM. The first term is to make the within-cluster distance as small as possible in subspace, and the second term is to allow more dimensions to participate in the clustering process. The parameter γ>0 controls the distribution of *w* in each dimension. The last term is a new between-cluster distance measure method proposed in this paper, which is to maximize the between-cluster distance. η>0 is a hyperparameter used to control the influence of between-cluster distance on the objective function, degenerating into the EWKM algorithm when η=0.

In order to optimize this function conveniently, it can modify Equation (12) as follows:

(13) $$\begin{array}{c} P\left(W,U,Z\right)=\left(1+\eta \right)\sum_{p=1}^{k}\sum_{i=1}^{n}u_{pi}\sum_{j=1}^{m}w_{j}{\left(x_{ij}-z_{pj}\right)}^{2}\\ +\gamma \sum_{j=1}^{m}w_{j}\log w_{j}-\eta \sum_{p=1}^{k}\sum_{i=1}^{n}\sum_{j=1}^{m}w_{j}{\left(x_{ij}-z_{pj}\right)}^{2}, \end{array}$$

subject to

$$\left\{\begin{array}{c} \sum_{j=1}^{m}w_{j}=1,\;\;0<w_{j}<1,\\ \sum_{p=1}^{k}u_{pi}=1,\;\;u_{pi}\in \left\{0,1\right\}. \end{array}\right.$$

It is established based on the three basic theorems below.

**Theorem** **1.** *Given U and Z are fixed, P is minimized only if:*

(14) $$w_{j}=\exp\left(\frac{-D_{j}}{\gamma }\right)/\sum_{t=1}^{m}\exp\left(\frac{-D_{t}}{\gamma }\right),$$

*where:*

(15) $$D_{j}=\left(1+\eta \right)\sum_{p=1}^{k}\sum_{i=1}^{n}u_{pi}{\left(x_{ij}-z_{pj}\right)}^{2}-\eta \sum_{p=1}^{k}\sum_{i=1}^{n}{\left(x_{ij}-z_{pj}\right)}^{2}.$$

**Proof.**  We use the Lagrangian multiplier technique to obtain the following unconstrained minimization problem:

(16) $$\begin{array}{c} \min P\left(\left\{w_{j}\right\},\left\{\alpha \right\}\right)=\left(1+\eta \right)\sum_{p=1}^{k}\sum_{i=1}^{n}u_{pi}\sum_{j=1}^{m}w_{j}{\left(x_{ij}-z_{pj}\right)}^{2}\\ -\eta \sum_{p=1}^{k}\sum_{i=1}^{n}\sum_{j=1}^{m}w_{j}{\left(x_{ij}-z_{pj}\right)}^{2}\\ +\gamma \sum_{j=1}^{m}w_{j}\log w_{j}+\alpha \left(\sum_{j=1}^{m}w_{j}-1\right) \end{array}.$$

where α is the Lagrange multiplier. By setting the gradient of the function Equation (16) with respect to $w_{j}$ and α to zero, we obtain the equations:

(17) $$\begin{array}{c} \frac{\partial P}{\partial w_{j}}=D_{j}+\gamma \left(\log w_{j}+1\right)+\alpha =0,\\ D_{j}=\left(1+\eta \right)\sum_{p=1}^{k}\sum_{i=1}^{n}u_{pi}{\left(x_{ij}-z_{pj}\right)}^{2}-\eta \sum_{p=1}^{k}\sum_{i=1}^{n}{\left(x_{ij}-z_{pj}\right)}^{2}, \end{array}$$

where $D_{j}$ includes the information of the within-cluster distance and the between-cluster distance of all the points on the dimension.

(18) $$\frac{\partial P}{\partial \alpha }=\sum_{j=1}^{m}w_{j}-1=0$$

From (17), we obtain:

(19) $$w_{j}=\exp\left(\frac{-D_{j}}{\gamma }\right)\exp\left(\frac{-\alpha -\gamma }{\gamma }\right).$$

Substituting (19) into (18), we have:

(20) $$\sum_{j=1}^{m}w_{j}=\exp\left(\frac{-\alpha -\gamma }{\gamma }\right)\sum_{j=1}^{m}\exp\left(\frac{-D_{j}}{\gamma }\right)=1.$$

From (20), we obtain:

(21) $$\exp\left(\frac{-\alpha -\gamma }{\gamma }\right)=1/\sum_{j=1}^{m}\exp\left(\frac{-D_{j}}{\gamma }\right).$$

Substituting (21) into (19), we have:

(22) $$w_{j}=\exp\left(\frac{-D_{j}}{\gamma }\right)/\sum_{t=1}^{m}\exp\left(\frac{-D_{t}}{\gamma }\right).$$

 □

**Theorem** **2.** *Given U and W are fixed, P is minimized only if:*

(23) $$z_{pt}=\frac{\left(1+\eta \right)\sum_{i=1}^{n}u_{pi}x_{it}-\eta \sum_{i=1}^{n}x_{it}}{\left(1+\eta \right)\sum_{i=1}^{n}u_{pi}-\eta n}$$

**Proof.**  When we have fixed *U* and *W*, from (13), we have:

(24) $$\begin{array}{c} Q\left(W,U,Z\right)=\left(1+\eta \right)\sum_{p=1}^{k}\sum_{i=1}^{n}u_{pi}\sum_{j=1}^{m}w_{j}{\left(x_{ij}-z_{pj}\right)}^{2}-\eta \sum_{p=1}^{k}\sum_{i=1}^{n}\sum_{j=1}^{m}w_{j}{\left(x_{ij}-z_{pj}\right)}^{2} \end{array}.$$

By setting the gradient of the function (24) with respect to $z_{pj}$ to zero, we obtain the equations:

(25) $$\begin{array}{c} \frac{\partial Q(W,U,Z)}{\partial z_{pj}}=2\left(1+\eta \right)\sum_{i=1}^{n}u_{pi}w_{j}\left(z_{pj}-x_{ij}\right)-2\eta \sum_{i=1}^{n}w_{j}\left(z_{pj}-x_{ij}\right)=0. \end{array}$$

From (25), we have:

(26) $$\left(1+\eta \right)\sum_{i=1}^{n}u_{pi}\left(z_{pj}-x_{ij}\right)=\eta \sum_{i=1}^{n}\left(z_{pj}-x_{ij}\right).$$

From (26), we derive:

(27) $$\sum_{i=1}^{n}u_{ip}z_{pj}+\eta \sum_{i=1}^{n}u_{pi}z_{pj}-\eta \sum_{i=1}^{n}z_{pj}=\sum_{i=1}^{n}u_{pi}x_{ij}+\eta \sum_{i=1}^{n}u_{pi}x_{ij}-\eta \sum_{i=1}^{n}x_{ij}.$$

From (27), we obtain:

(28) $$z_{pj}\left[\left(1+\eta \right)\sum_{i=1}^{n}u_{pi}-\eta n\right]=\left(1+\eta \right)\sum_{i=1}^{n}u_{pi}x_{ij}-\eta \sum_{i=1}^{n}x_{ij}.$$

then, from (28) we have (23). □

**Theorem** **3.** *Similarly to the k-means algorithm, given Z and W are fixed, u is updated as:*

(29) $$u_{pi}=\left\{\begin{array}{c} 1,\;if\;\;\sum_{j=1}^{m}w_{j}{\left(x_{ij}-z_{pj}\right)}^{2}\geq \sum_{j=1}^{m}w_{j}{\left(x_{ij}-z_{p^{\prime }j}\right)}^{2}\\ 0,\;otherwise \end{array}\right..$$

The detailed proof process about Theorem 3 can be found in [34,35].

The ERKM algorithm that minimizes Equation (12), using (14), (23), and (29), is summarized as follows (Algorithm 1):

**Algorithm 1** ERKM.**Input**: The number of clusters *k* and parameters γ,η; Randomly choose *k* cluster centers, and set all initial weights with a normalized uniform distribution;  **repeat** Fixed Z,W, update the partition matrix *U* by (29)  Fixed U,W, update the cluster centers *Z* by (23)  Fixed U,Z, update the dimension weights *W* by (14)  **until** Convergence  **return**
U,Z,W

The hyperparameter η is used to balance the within-cluster distance and the between-cluster distance. It has the following features in the control of the clustering process:

- When $0<\eta <\frac{\sum_{p=1}^{k}\sum_{i=1}^{n}u_{pi}{\left(x_{ij}-z_{pj}\right)}^{2}}{\sum_{p=1}^{k}\sum_{i=1}^{n}\left(1-u_{pi}\right){\left(x_{ij}-z_{pj}\right)}^{2}}$, according to (14) and (15), $w_{j}$ is inversely proportional to $D_{j}$. The more important the *j*-th dimension, the larger $w_{j}$, and the smaller $D_{j}$.
- For others, according to (14) and (15), $w_{j}$ is proportional to $D_{j}$. The larger $D_{j}$, the larger $w_{j}$. This violates the basic idea that the more important the corresponding dimension, the smaller the sum of the distance on this dimension. Under this circumstance, it will cause the value of $D_{j}\leq 0$, so that the objective function diverges.

**Proof.**  According to (14), if we want to satisfy this basic idea, namely the smaller $D_{j}$, the larger $w_{j}$, $D_{j}$ should be great than zero. Hence, from (15), we have:

(30) $$D_{j}=\left(1+\eta \right)\sum_{n=1}^{k}\sum_{i=1}^{n}u_{pi}{\left(x_{ij}-z_{pj}\right)}^{2}-\eta \sum_{n=1}^{k}\sum_{i=1}^{n}{\left(x_{ij}-z_{pj}\right)}^{2}>0.$$

From (30), we obtain:

(31) $$\sum_{p=1}^{k}\sum_{i=1}^{n}u_{pi}{\left(x_{ij}-z_{pj}\right)}^{2}-\eta \sum_{p=1}^{k}\sum_{i=1}^{n}\left(1-u_{pi}\right){\left(x_{ij}-z_{pj}\right)}^{2}>0.$$

Moreover, according to (31), we have:

(32) $$\sum_{p=1}^{k}\sum_{i=1}^{n}u_{pi}{\left(x_{ij}-z_{pj}\right)}^{2}>\eta \sum_{p=1}^{k}\sum_{i=1}^{n}(1-u_{pi}){\left(x_{ij}-z_{pj}\right)}^{2}.$$

Finally, from (32), we derive:

(33) $$\eta <\frac{\sum_{p=1}^{k}\sum_{i=1}^{n}u_{pi}{\left(x_{ij}-z_{pj}\right)}^{2}}{\sum_{p=1}^{k}\sum_{i=1}^{n}(1-u_{pi}){\left(x_{ij}-z_{pj}\right)}^{2}}.$$

 □

### 3.2. Convergency and Complexity Analysis

For the ERKM algorithm, when the parameter η satisfies this condition (33), global or local optimal values will be obtained after a finite number of iterations. Obviously, there are only a finite number of possible partitions *U*, because the number of points is not infinite, and each of the possible partitions will appear only once in the clustering process. Similar to [16], assume that we have $U^{t_{1}}=U^{t_{2}}$, where $t_{1}\neq t_{2}$ and $t_{i}$ represents the number of iterations. Then, based on $U^{t_{i}}$, we can obtain $Z^{t_{i}}$ by minimizing $Q(W,U^{t_{i}},Z)$ according to (24). Subsequently, $Z^{t_{1}}$ and $Z^{t_{2}}$ are obtained respectively, and furthermore, $Z^{t_{1}}=Z^{t_{2}}$ because $U^{t_{1}}=U^{t_{2}}$. Finally, according to (14), we can compute the minimizer $W^{t_{1}}$ and $W^{t_{2}}$ by using $U^{t_{1}}$ and $Z^{t_{1}}$, and $U^{t_{2}}$ and $Z^{t_{2}}$ respectively. Naturally, $W^{t_{1}}=W^{t_{2}}$ again. Therefore, we obtain $P\left(U^{t_{1}},Z^{t_{1}},W^{t_{1}}\right)=P\left(U^{t_{2}},Z^{t_{2}},W^{t_{2}}\right)$. However, the sequence P(∗,∗,∗) is strictly decreasing, which is broken by the analysis result, that is to say, the ERKM algorithm converges in a finite number of iterations.

Similar to the basic *k*-means algorithm, the proposed one is also iterative. The computational complexity of the basic *k*-means is O(tmnk), where *t* is the iterative times; m,n, and *k* are the number of dimensions, points, and clusters, respectively. As shown in Section 3.1, ERKM has three computational steps including updating the weights, updating the cluster centers, and updating the partition matrix [16]. The complexity of updating the weights is O(kmn). The complexities of updating the cluster centers and partition matrix are O(kmn+mn+1) and O(kmn), respectively. Hence, the overall computational complexity of ERKM is also O(tkmn). Compared with the basic *k*-means algorithm, its only needs extra O(kmn) computational time to calculate the weights and O(mn+1) to calculate the distance of each point *i* in dimension *j*. Fortunately, it does not change the total computational complexity of ERKM.

## 4. Experiments and Discussion

### 4.1. Experimental Setup

In the experiments, the performance of the proposed algorithm was extensively evaluated on two synthetic and seven real-life datasets tabulated in Table 1, which can be downloaded at the UCI website. We compared the clustering results produced by ERKM with the benchmark clustering algorithms including basic *k*-means (KMEA), WKME, EWKM, ESSC, and the last three years’ clustering algorithms, AFKM [36] Sampling-Clustering (SC) [37], and SSC-MP [38].

As we all know, most of *k*-means-type clustering algorithms produce local optimal solution, and the final results depend on the initial cluster centers. For the weighting *k*-means algorithms: WKME and EWKM (also including ours), the initial weights also affect the final results. For the sake of fairly comparing the clustering results, all the cluster centers and weights of each algorithm were randomly initialized. Finally, we compared the average value and standard deviation of each metric produced by the algorithms after 100 runs. in order to speed up the convergence time of the algorithms, all datasets were normalized.

### 4.2. Evaluation Method

In this paper, four metrics, Accuracy (Acc), Adjusted Rand Index (ARI), F-score (Fsc), and Normal Mutual Information (NMI), were used for evaluating the proposed ERKM algorithm. Acc was used to measure the accuracy of clustering results, and its value range was [0, 1]. Fsc is a weighted harmonic average of precision and recall, and its value range was also [0, 1]. Both ARI and NMI were used to measure the degree of agreement between the two data distributions, which ranged from [−1, 1] and [0, 1], respectively. For the above four evaluation metrics, the larger the value, the better the clustering results. More detail can be found in [19].

### 4.3. Parameter Setting

Form the objective function (12), the proposed algorithm had only two hyperparameters γ and η. Hence, we will first choose proper parameters for γ and η produced by the results on the datasets.

- Parameter γ:
  Hyperparameter γ appears in the EWKM, ESSC, and ERKM algorithms. ESSC and ERKM algorithms were directly extended from the EWKM algorithm, and only a between-cluster distance constraint was added to the objective function of the EWKM algorithm. At the same time, since the EWKM only contained one parameter γ, the value of γ can be studied by the performance of EWKM on two synthetic and seven real-life datasets, respectively. We fixed the range of γ in [1, 50] and set the step to 1.
- Parameter η:
  In the algorithm ERKM, η has a similar effect as α in the ESSC algorithm. However, since the two algorithms adopted different feature weighting methods and between-cluster distance measures, a reasonable η value will be selected by the results of the ERKM algorithm on two synthetic and seven real-life datasets, respectively. Since the within-cluster distance and the between-cluster distance have different contributions to the whole clustering process, the value of η should satisfy the condition (33), so as to avoid the divergence of the objective function. In the section below, we fixed the range of η in [0.002, 0.2] and set the step to 0.002 in [0, 0.05] and 0.02 in (0.05, 0.2] to search for a proper value.

In summary, we will first choose parameter γ by the results of EWKM and parameter η by the results of ERKM both on two synthetic and seven real-life datasets. For the rest of the comparative algorithms, the default configuration was done (as tabulated in Table 2).

### 4.4. Experiment on Synthetic Data

In this section, we first use two synthetic datasets as described in [27] to verify the performance of the proposed algorithm ERKM. In each synthetic dataset, there were three clusters.

**Figure 3:**
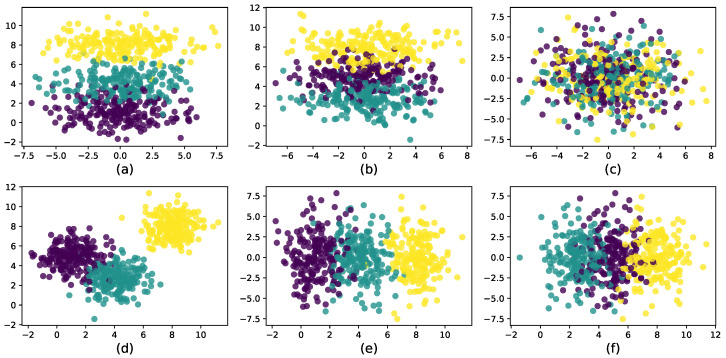
Synthetic dataset with three clusters in the two-dimensional subspace of $x_{2},x_{3}$ and two noise dimensions $x_{1},x_{4}$. (**a**) Subspace of $x_{1},x_{2}$. (**b**) Subspace of $x_{1},x_{3}$. (**c**) Subspace of $x_{1},x_{4}$. (**d**) Subspace of $x_{2},x_{3}$. (**e**) Subspace of $x_{2},x_{4}$. (**f**) Subspace of $x_{3},x_{4}$.

For the first synthetic dataset: there was a total of 4 variables with 2 effective, while the other 2 were noise variables. There were 500 points scattered in three clusters with 200 in the first cluster, 100 in the second cluster, and 200 in the last one. As plotted in Figure 3, each dimension was independent and identically distributed (iid) from a normal distribution with the 2 effective features iid from N(5,1) and N(1,1), respectively, for Cluster 1, N(2.5,1) and N(4.0,1), respectively, for Cluster 2, and N(8,1) for Cluster 3; the remaining 2 features were all iid from N(0,1) for all clusters.

For the second synthetic dataset: there was a total of 1000 variables with 150 effective, while the other 850 were noise. Specifically, there were 250 points with 100 in Cluster 1, 50 in Cluster 2, and 100 in the other. The 150 effective features were all iid from N(0,1) for the first cluster, N(1.5,1) for the second cluster, and N(2,1) for the third cluster; the remaining 850 features were all iid from N(0,1) for the three clusters.

**Figure 4:**
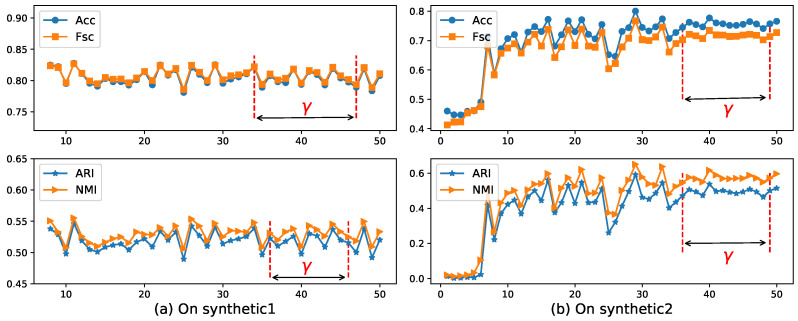
Four metrics’ change with EWKM for different γ on two synthetic datasets. Acc: accuracy; Fsc: F-score; ARI: adjusted rand index; NMI: normal mutual information.

Now, we use the EWKM algorithm on two synthetic datasets to select a reasonable value of parameter γ using the four metrics selected in Section 4.2. Figure 4 shows the change of Acc, Fsc, ARI, and NMI for EWKM on synthetic datasets.

#### 4.4.1. Parameter Study

**Figure 5:**
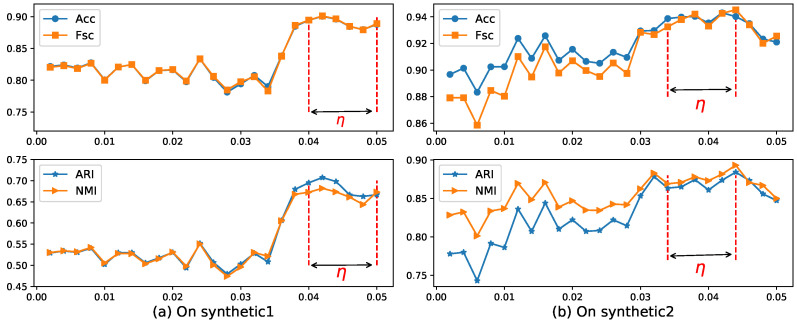
Four metrics’ change with ERKM for different η on two synthetic datasets.

As can be seen from Figure 4, when the value of γ was located in the range of 36–49 on Synthetic 1 and 36–45 on Synthetic 2, the algorithm EWKM was generally stable, where the changes in the four metrics were within 2%. However, in the other range, the performance of EWKM was greatly affected by γ, either greater than about 4% or no convergence. from Figure 5, we can see that when the range of η was about 0.04–0.05 on Synthetic 1 and 0.034–0.044 on Synthetic 2, the results of ERKM with a higher performance did not change much. Therefore, on these two synthetic datasets, we chose the two hyperparameters γ=40.0,η=0.04.

#### 4.4.2. Results and Analysis

**Figure 6:**
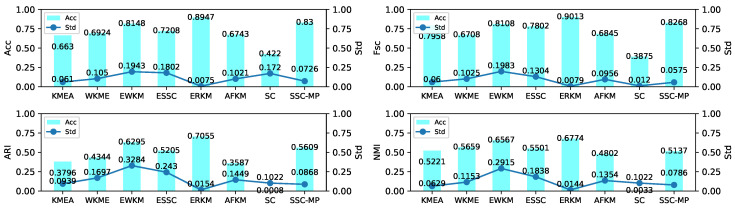
Results of the eight algorithms on the Synthetic 1 dataset.

**Figure 7:**
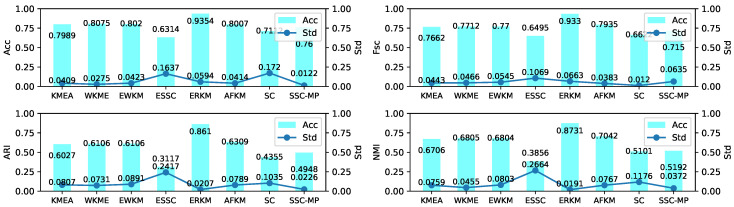
Results of the eight algorithms on the Synthetic 2 dataset.

The experiment results shown in Figure 6 and Figure 7 on two synthetic datasets indicated that: (1) the Acc, Fsc, ARI, and NMI of the ERKM algorithm were higher than v other algorithms; ERKM achieved the highest score on the four metrics on both datasets. In Acc and Fsc, the results of ERKM were at least 6% higher than that of the second-best algorithm on Synthetic 1 and at least 13% on Synthetic 2. In ARI and NMI, it also was 2% higher than that of the second on Synthetic 1. Compared with the other algorithms, ERKM obtained significant achievement on Synthetic 2, where each of metric was 13% higher than that of the second-best, especially in ARI and NMI by more than 17%. (2) From the perspective of stability, ERKM, achieved the best one of all eight algorithms. From the standard deviation, ERKM achieved the minimum value among the four metrics on Synthetic 1 and good results on Synthetic 2.

### 4.5. Experiment on Real-Life Data

#### 4.5.1. Parameter Study

**Figure 8:**
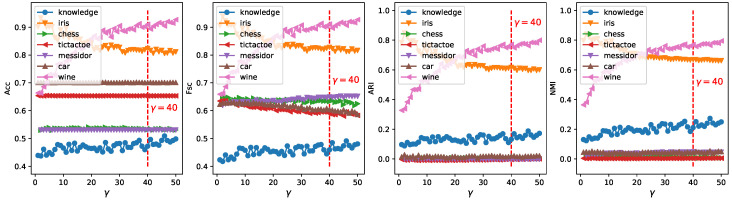
Four metrics’ change with EWKM for different γ on real-life datasets.

In this section, we will also use the EWKM algorithm on seven real-life datasets to select a reasonable parameter γ with the four metrics selected in Section 4.2. Figure 8 shows the change of four metrics of EWKM on seven real-life datasets.

It can be seen from Figure 8 that among the four metrics, EWKM had a large fluctuation mainly concentrating on the two datasets of iris and wine, and also a little fluctuation on knowledge. However, there was almost no significant change on the remaining four datasets. Therefore, this paper only determines the value of γ from the results of EWKM on the three datasets of iris, wine, and knowledge.

In Figure 8, the results of EWKM on the dataset wine changed by more than 30% for all four metrics and more than 40% for ARI and NMI. At the same time, with the increase of γ, the results of EWKM on the dataset wine was increasing and generally tended to be stable near γ=40, with a change of less than 2%. On the iris dataset, the results of EWKM on all four metrics showed a downward trend with a change of more than 10% and generally stabilized after γ>30. Finally, for the dataset knowledge, the EWKM algorithm had a small change for the four metrics, mainly within 10%, and with the increase of γ, the results of EWKM slightly increased, while when γ was around 40, it tended to be stable overall. In summary, through the above comparison and analysis, we found that for the two algorithms EWKM and ESSC, when γ was equal to 40, it was a reasonable value.

**Figure 9:**
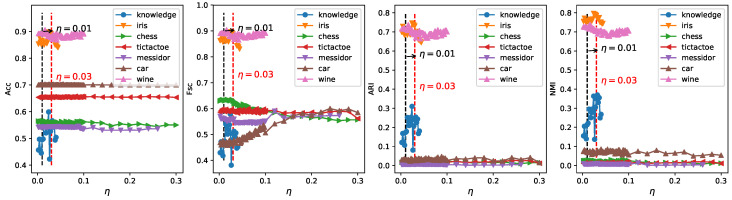
Four metrics’ change with ERKM for different η on real-life datasets.

In order to obtain a robust range of eta, which should enable ERKM to have reasonable results for most unknown datasets, we can identify it by analyzing the results of ERKM on seven known real-life datasets. Figure 9 is a plot of the results of ERKM on seven real-life datasets. Then, we will determine a reasonable η value by analyzing the results in Figure 9. From the three metrics of Acc, ARI, and NMI, with the increase of η, the results of the ERKM algorithm showed a significant change only for the three datasets wine, iris, and knowledge. For the dataset wine, with the increase of η, the results of ERKM started to decrease slightly around η=0.01 and tended to be stable in the vicinity of η=0.03. For the dataset iris, the results of ERKM, from η=0.01, started to rise and were stable at around η=0.03. For the dataset knowledge, when 0.01<η≤0.03, the results of ERKM were generally in a stable state.

For Fsc, when η>0.03, the results of ERKM on the datasets wine, iris, knowledge, and chess had a declining trend and on the dataset car had an increasing trend, while they were almost unchanged on tictactoe and messidor. When η<0.01, the results had large fluctuations only on knowledge; and when 0.01<η≤0.03, the results on the seven datasets were generally stable.

In summary, through the above analysis of the ERKM algorithm on the four metrics, it had a reasonable value for η=0.03.

#### 4.5.2. Results and Analysis

In this section, we will compare ERKM (with γ=40.0 and η=0.03 obtained in Section 4.5.1) with all the algorithms on seven real-life datasets and analyze the results. The final test results are tabulated in Table 3.

As can be seen from Table 3, for Acc, the results of ERKM for the five datasets were higher than the other seven algorithms. Among them, the results on knowledge and iris were the best, which was 6% higher than the second-best algorithm ESSC. On datasets tictactoe, chess, and messidor, it was also higher than the second-best algorithm by 0.41%, 2.8%, and 0.4%, respectively. For Fsc, ERKM performed better than the other seven algorithms on the four datasets: 1.44% higher than the second-best algorithm for the dataset knowledge, 5.38% higher for iris, 0.14% higher for tictactoe, and 4% higher for car. For ARI, the results of the ERKM algorithm on the six datasets were higher than the other seven algorithms: for the datasets knowledge, iris, tictactoe, chess, messidor, and wine, it was higher than the second-best algorithm by 8.05%, 13.2%, 1.23%, 1.21%, 0.2%, and 1.12%, respectively. For NMI, the results of the ERKM algorithm were higher than the other seven algorithms on the four datasets: 13.3% higher than the second-best algorithm for the dataset iris, 13.4% higher for knowledge, 0.93% higher for tictactoe, and 0.14% higher for chess.

**Figure 10:**
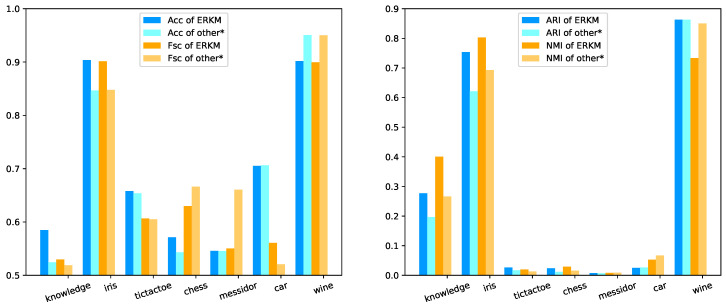
The results of ERKM and others* on real-life datasets. other* means the second-best algorithm, if ours is the best; or the best, if ours is not the best.

From Figure 10 left, it is clear that ERKM obtained in general better results than the other second-best cluster algorithms on most datasets. Just as clearly, for the metrics of Acc and Fsc, the results of ERKM were higher than the second-best algorithms on the datasets knowledge, iris, and tictactoe; and nearest to the best algorithms on the datasets chess, messidor, and car. On the right, it shows that the results of ERKM were much better than the others on the datasets knowledge and iris. On the rest of the datasets, the results of ERKM were slightly better than others.

Based on the two-part analysis above, the ERKM algorithm could be better than the other seven clustering algorithms in most cases. That is to say, through the experimental results, the new between-cluster distance measure can effectively improve the clustering results.

### 4.6. Convergence Speed

**Figure 11:**
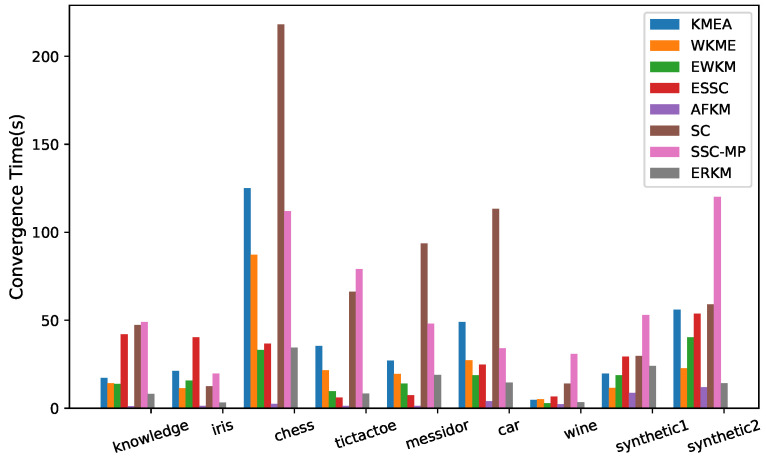
Convergence time.

Figure 11 shows the convergence time of the eight algorithms on the two synthetic and seven real-life datasets. The method of recording convergence time was to record the total time spent by each algorithm run on each dataset 100 times with randomly initializing the cluster center and weights. It can be seen from Figure 11 that in addition to AFKM, on the six datasets of knowledge, iris, chess, car, wine, and Synthetic 2, the convergence speed of the ERKM algorithm was significantly faster than that of the other five algorithms. It was also relatively moderate on the other three datasets, tictactoe, messidor, and Synthetic 1. Compared with the algorithm KMEA, WKME, EWKM, ESSC, SC, and SSC-MP, the convergence speed of the algorithm ERKM was the best one on the datasets knowledge, iris, car, and wine. The reason may be that the KMEA, WKKM, and EWKM algorithms did not utilize the between-cluster distance, and WKME and EWKM used complex matrix feature weighting. Although the ESSC algorithm incorporated the between-cluster information, its measure was improper. for the algorithm SC, before clustering, it needed to generate a KNN-graph. In general, ERKM had a good and competitive convergence speed.

### 4.7. Robustness Analysis

The two hyperparameters γ and η of the algorithm ERKM will affect the performance of the algorithm. At the same time, the ERKM is directly extended from the EWKM where γ is used to control the effect of weights on the objective function. From Figure 4 and Figure 8, it is clear that when γ was around 40, the results of the algorithm ERKM were relatively stable on both synthetic data and real-life data. Then, we fixed γ=40.0 to analyze the effect of η on ERKM. In ERKM, η plays the role of adjusting the influence between within-cluster distance and between-cluster distance. Therefore, the robustness of the algorithm ERKM can be analyzed by its sensitivity to hyperparameters η. From Figure 5 and Figure 9, when η was around 0.03, the algorithm ERKM was relatively stable on both synthetic and real-life datasets.

## 5. Conclusions

In this paper, a new soft subspace clustering algorithm based on between-cluster distance and entropy regularization was proposed. Different from the traditional algorithms that utilize the between-cluster information by maximizing the distance between each cluster center and the global center (e.g., ESSC), ERKM effectively uses the between-cluster information by maximizing the distance between the points in the subspace that do not belong to the cluster and the center point of the cluster. Based on this assumption, this paper first designed an objective function for the algorithm and then derived the update formula by the Lagrange multiplier method. Finally, we compared several traditional subspace clustering algorithms on seven real datasets and concluded that the ERKM algorithm can achieve better clustering results in most cases.

In real-world applications, many high-dimensional data have various cluster structure features. In future research work, we plan to expand the application scope of the algorithm by modifying the objective function to adapt the case of including various complex cluster structures.

## Figures and Tables

**Table 1:** Information of the datasets.

Datasets	Points	Dimensions	Clusters
Synthetic1	500	4	3
Synthetic2	250	1000	3
Knowledge	403	5	4
Iris	150	4	3
Chess	3196	36	2
Tictactoe	958	9	2
Messidor	1151	19	2
Car	1728	6	4
Wine	178	13	3

**Table 2:** Default parameters of comparative algorithms. * We will study this parameter of the corresponding algorithms.

Algorithms	Parameters
KMEA	-
WKME [10]	β=7
EWKM [16]	γ=∗
ESSC [5]	γ=∗,α= 0.01
AFKM [36]	-
SC [37]	r = 0.2, size = 16
SSC-MP [38]	$s_{max}=3,p_{max}=none,\tau =100$

**Table 3:** Results on real-life datasets (standard deviation in brackets).

Metric	Model	Knowledge	Iris	Tictactoe	Chess	Messidor	Car	Wine
	KMEA	0.4600(0.02)	0.8054(0.06)	0.6535(0.01)	0.5359(0.02)	0.5452(0.01)	0.7057(0.02)	0.9443(0.07)
	WKME	0.4897(0.06)	0.7847(0.08)	0.6534(0.00)	0.5432(0.03)	0.5382(0.01)	0.7063(0.016)	0.9471(0.07)
	EWKM	0.4789(0.09)	0.8209(0.06)	0.6534(0.00)	0.5334(0.02)	0.5329(0.00)	0.7011(0.01)	0.9024(0.08)
Acc	ESSC	0.5244(0.02)	0.8466(0.00)	0.6539(0.00)	0.5288(0.00)	0.5308(0.00)	0.7004(0.00)	0.9506(0.00)
	AFKM	0.4780(0.04)	0.8127(0.06)	0.6534(0.00)	0.5354(0.00)	0.5416(0.01)	0.7002(0.00)	0.9399(0.08)
	SC	0.4987(0.00)	0.8066(0.00)	0.6535(0.00)	0.5222(0.00)	0.5308(0.00)	0.7002(0.01	0.8707(0.00)
	SSC-MP	0.5012(0.00)	0.7120(0.01)	0.6513(0.00)	0.5222(0.00)	0.5311(0.00)	0.7002(0.00)	0.5865(0.00)
	ERKM	0.5848(0.08)	0.9036(0.01)	0.6580(0.01)	0.5714(0.03)	0.5456(0.01)	0.7051(0.02)	0.9016(0.04)
	KMEA	0.1945(0.02)	0.8115(0.04)	0.5672(0.03)	0.5270(0.02)	0.5533(0.03)	0.4613(0.06)	0.9469(0.06)
	WKME	0.4769(0.05)	0.7979(0.07)	0.5643(0.03)	0.5350(0.03)	0.6029(0.06)	0.4551(0.05)	0.9482(0.06)
	EWKM	0.4652(0.09)	0.8264(0.05)	0.6051(0.03)	0.6210(0.05)	0.6601(0.01)	0.5206(0.06)	0.9046(0.07)
Fsc	ESSC	0.5151(0.03)	0.8477(0.00)	0.6004(0.04)	0.6642(0.00)	0.6604(0.01)	0.5156(0.05)	0.9503(0.00)
	AFKM	0.4689(0.03)	0.8158(0.05)	0.5699(0.03)	0.5895(0.00)	0.5814(0.05)	0.3864(0.04)	0.9431(0.06)
	SC	0.4355(0.21)	0.4719(0.00)	0.5709(0.01)	0.543(0.00)	0.5349(0.00)	0.3818(0.04)	0.8694(0.00)
	SSC-MP	0.5189(0.01)	0.7672(0.00)	0.5207(0.00)	0.6662(0.00)	0.5629(0.00)	0.5176(0.00)	0.5842(0.00)
	ERKM	0.5295(0.08)	0.9015(0.01)	0.6065(0.04)	0.6297(0.02)	0.5503(0.02)	0.5606(0.06)	0.8997(0.04)
	KMEA	0.1318(0.02)	0.5890(0.06)	0.0140(0.02)	0.0068(0.01)	0.0070(0.00)	0.0526(0.05)	0.8580(0.11)
	WKME	0.1588(0.06)	0.5719(0.10)	0.0128(0.02)	0.0113(0.01)	0.0024(0.00)	0.0474(0.04)	0.8632(0.11)
	EWKM	0.1425(0.14)	0.6144(0.07)	−0.0028(0.02)	0.0047(0.01)	0.0000(0.00)	0.0152(0.05)	0.7545(0.12)
ARI	ESSC	0.1965(0.03)	0.6210(0.00)	0.0023(0.02)	0.0015(0.00)	−0.0008(0.00)	0.0259(0.05)	0.8520(0.00)
	AFKM	0.1412(0.04)	0.5987(0.07)	0.0171(0.02)	0.0053(0.00)	0.0046(0.00)	0.0162(0.02)	0.8496(0.12)
	SC	0.1062(0.00)	0.1851(0.00)	0.0123(0.00)	0.0004(0.00)	0.0013(0.00)	−0.0070(0.02)	0.6458(0.00)
	SSC-MP	0.1453(0.00)	0.5520(0.00)	0.0055(0.00)	0.0013(0.00)	0.0020(0.00)	0.0266(0.00)	0.2135(0.00)
	ERKM	0.277(0.10)	0.7535(0.01)	0.0263(0.05)	0.0234(0.02)	0.0072(0.00)	0.0247(0.01)	0.8632(0.11)
	KMEA	0.1945(0.02)	0.6472(0.02)	0.0100(0.01)	0.0054(0.01)	0.0088(0.01)	0.1187(0.06)	0.8474(0.08)
	WKME	0.2323(0.08)	0.6511(0.04)	0.0084(0.01)	0.0091(0.01)	0.0184(0.01)	0.0962(0.06)	0.8503(0.08)
	EWKM	0.2197(0.15)	0.6691(0.02)	0.0038(0.00)	0.0074(0.01)	0.0261(0.01)	0.0396(0.03)	0.7620(0.09)
NMI	ESSC	0.2662(0.03)	0.6321(0.00)	0.0060(0.011)	0.0153(0.01)	0.0089(0.01)	0.0458(0.04)	0.8197(0.00)
	AFKM	0.2102(0.05)	0.6560(0.03)	0.0127(0.01)	0.0125(0.00)	0.0164(0.01)	0.0431(0.03)	0.8414(0.09)
	SC	0.1819(0.00)	0.4667(0.00)	0.0045(0.01)	0.0003(0.00)	0.0011(0.00)	0.0159(0.04)	0.649(0.00)
	SSC-MP	0.1684(0.00)	0.6930(0.01)	0.0032(0.00)	0.0006(0.00)	0.0087(0.00)	0.0671(0.00)	0.2289(0.00)
	ERKM	0.4003(0.14)	0.8026(0.01)	0.0193(0.03)	0.0291(0.03)	0.0079(0.00)	0.0526(0.06)	0.7333(0.05)

## Abbreviations

The following abbreviations are used in this manuscript:

ERKM	Entropy Regularization *k*-means
KMEA	*k*-Means
WKME	Weighted *k*-Means
EWKM	Entropy Weighted *k*-Means
ESSC	Enhanced Soft-Subspace Clustering
AFKM	Assumption-Free *k*-Means
SC	Sampling-Clustering
SSC-MP	Subspace Sparse Clustering by the greedy orthogonal Matching Pursuit
Acc	Accuracy
ARI	Adjusted Rand Index
Fsc	F-score
NMI	Normal Mutual Information
